# Using vignettes to assess contributions to the work of addressing child mental health problems in primary care

**DOI:** 10.1186/s12913-015-1237-x

**Published:** 2016-01-22

**Authors:** Lawrence S. Wissow, Waleed Zafar, Kate Fothergill, Anne Ruble, Eric Slade

**Affiliations:** Department of Health, Behavior, and Society, Johns Hopkins School of Public Health, 703 Hampton House, 624 N. Broadway, Baltimore, MD USA; Department of Emergency Medicine, World Health Organization Collaborating Center for Emergency Medicine and Trauma Care, Aga Khan University, Karachi, Pakistan; Department of Psychiatry, Johns Hopkins School of Medicine, Baltimore, MD USA; Department of Psychiatry, University of Maryland School of Medicine, Baltimore, MD USA

**Keywords:** Children, Mental health, Primary care, Collaborative care, Task shifting, Vignettes, Survey

## Abstract

**Background:**

To further efforts to integrate mental health and primary care, this study develops a novel approach to quantifying the amount and sources of work involved in shifting care for common mental health problems to pediatric primary care providers.

**Methods:**

Email/web-based survey of a convenience sample (*n* = 58) of Maryland pediatricians (77 % female, 58 % at their site 10 or more years; 44 % in private practice, 52 % urban, 48 % practicing with a co-located mental health provider). Participants were asked to review 11 vignettes, which described primary care management of child/youth mental health problems, and rate them on an integer-based ordinal scale for the overall amount of work involved compared to a 12th reference vignette describing an uncomplicated case of ADHD. Respondents were also asked to indicate factors (time, effort, stress) accounting for their ratings. Vignettes presented combinations of three diagnoses (ADHD, anxiety, and depression) and three factors (medical co-morbidity, psychiatric co-morbidity, and difficult families) reported to complicate mental health care. The reference case was pre-assigned a work value of 2. Estimates of the relationship of diagnosis and complicating factors with workload were obtained using linear regression, with random effects at the respondent level.

**Results:**

The 58 pediatricians gave 593 vignette responses. Depression was associated with a 1.09 unit (about 50 %) increase in work (95 % CL .94, 1.25), while anxiety did not differ significantly from the reference case of uncomplicated ADHD (*p* = .28). Although all three complicating factors increased work ratings compared with the reference case, family complexity and psychiatric co-morbidity did so the most (.87 and 1.07 units, respectively, *P* < .001) while medical co-morbidity increased it the least (.44 units, *p* < .001). Factors most strongly associated with increased overall work were physician time, physician mental effort, and stress; those least strongly associated were staff time, physician physical effort, and malpractice risk. Pediatricians working with co-located mental health providers gave higher work ratings than did those without co-located staff.

**Conclusions:**

Both diagnosis and cross-diagnosis complicating factors contribute to the work involved in providing mental health services in primary care. Vignette studies may facilitate understanding which mental health services can be most readily incorporated into primary care as it is presently structured and help guide the design of training programs and other implementation strategies.

**Electronic supplementary material:**

The online version of this article (doi:10.1186/s12913-015-1237-x) contains supplementary material, which is available to authorized users.

## Background

World-wide, anxiety and depression are the most common mental health problems [1]. Both can occur during childhood and adolescence, often as the first manifestations of what will be lifetime difficulties [2]. There is evidence that it is possible to reduce the incidence of these conditions, [3–6] and that treatment during childhood can reduce some of their long-term adverse consequences on family, school, and peer functioning [7–9]. However, by some estimates, fewer than 20 % of children and youth who develop a mental health problem receive care, [10] and even in highly resourced countries, child mental health services are in short supply [11].

Better integration of mental health into primary medical care services has been proposed as a core approach to building mental health treatment capacity worldwide [12]. Integration involves both task shifting (primary care providing some mental health services rather than limiting its role to detection and referral) and improved collaboration between primary care providers and mental health specialists [13, 14]. Task shifting has emerged as particularly important, even in communities where mental health resources are available: not all families are willing to begin treatment in a specialty setting, and not all problems are severe enough for referral at the point at which they first emerge [15].

In the US, attempts to promote task shifting for child mental health problems in primary care have faced barriers related to pediatricians’ lack of confidence in their skills and sense of the additional work that would be involved [16, 17]. However, treatment of attention deficit/hyperactivity disorder (ADHD) has been an exception. ADHD has been broadly embraced by US pediatricians, in part because it is perceived as being easy to diagnose and efficiently treated with medication in a manner similar to common child somatic problems [18]. Other child mental health problems are seen as involving more work because they are harder to diagnose and require treatment based more on psychosocial interventions, which entails the need for additional skills and time [19].

Supporters of task shifting propose addressing these barriers by designing psychosocial interventions that are both customized for use in primary care and effective across multiple diagnoses [20–22]. They reason that primary care providers might be more likely to take on mental health work if they could master a relatively small set of cross-cutting diagnostic and treatment skills that would help them efficiently decide what first-line treatment to offer or reach agreement with a family about the need for a referral.

While some cross-cutting skills might involve treatments that target problems involved in many diagnoses (for example, advice about parent–child interaction is useful for anxiety, oppositional behavior, and ADHD), other skills might target situations that arise during evaluation and treatment planning. In qualitative studies [23, 24] and work preparing for the study reported here, we found three major issues that contributed to primary care providers’ sense of the work involved to treat mental health problems, regardless of the diagnosis: differentiating mental health problems from overlapping somatic problems, deciding what to treat when multiple mental health diagnoses were suspected, and working with “difficult” families (which included elements of family stress/dysfunction and a strained relationship with the provider). Our goal in the research reported here was to estimate the extent to which addressing these cross-cutting evaluation issues could reduce pediatricians’ reluctance to provide initial management of mental health problems beyond ADHD. To develop this estimate we turned to the use of clinical vignettes. Vignette-based ratings have been used to measure provider attitudes toward various forms of medical care [25] and are capable of reflecting the relationship of particular patient characteristics with providers’ actual clinical decision-making [26]. Vignettes were also used to develop the comparative work estimates (“relative value units”) on which physician payments are based in the US [27].

In the present study, we asked practicing pediatricians to provide assessments of the work involved in caring for children with three broad areas of mental health problems – ADHD, anxiety, and depression. The pediatricians were presented with a series of vignettes that systematically varied both diagnosis and patient/family characteristics corresponding to the cross-cutting issues identified in our qualitative research. We hypothesized that accounting for the cross-cutting issues would reduce or eliminate differences in work attributable to diagnosis.

Our analyses also sought to address a second issue related to task shifting – whether pediatricians saw the extra work involved in mental health care as falling primarily on themselves or on members of their office staff. Many adult and child mental health integration programs involve task shifting from off-site specialty mental health providers to social workers or “mid-level” therapists working at the primary care site (who may be employed by the practice or co-located but employed independently or by a community mental-health agency) [14]. We hypothesized that pediatricians working with co-located mental health professionals would give the vignettes lower work ratings or indicate that any increased work would fall on staff rather than on themselves.

## Methods

### Vignettes

We developed 12 vignettes, four each evoking symptoms suggestive of ADHD, anxiety, and depression. The core symptom descriptions were derived from cases described by participants in our original qualitative work [23, 24]. We adjusted them so that their level of severity would be more than a “developmental variation” (something likely addressed by monitoring rather than an intervention) but less than that which would trigger an urgent specialist evaluation [28]. Thus, the vignettes avoided situations in which the provider might be tempted to simply reassure the parent or immediately refer (for example, marked impairment of function, likely victimization, or suicidal ideation). Themes from the interviews were reviewed and refined at a meeting of community pediatricians (from the Community Advisory Committee of the Johns Hopkins Center for Mental Health in Pediatric Primary Care). The same group later reviewed drafts of the vignettes to check on their similarity to commonly seen situations. Subsequently 12 additional community pediatricians anonymously rated a revised set of vignettes, indicating the relative amount of work involved in managing similar cases and whether the vignettes reflected the range of work they commonly encountered. These responses were used to create the final versions of the vignettes and response questions used in the study. For each diagnosis, one vignette presented a description of an uncomplicated case (no diagnostic uncertainty and family amenable to treatment) and three vignettes described cases involving, respectively, possible somatic co-morbidity, possible mental health co-morbidity, or a difficult family (Table 1).

**Table 1:** Root/uncomplicated vignettes and additions for cross-cutting factors

Diagnosis	Root vignette	Cross-cutting factor	Text excerpts
ADHD	An 8 year-old boy, who has been a patient of yours for several years, has no known developmental issues, no chronic health problems, and lives with a stable, well-functioning family. He has long had moderate academic difficulty in school despite good effort; today he comes with his mother who has brought a packet of Vanderbilt forms (mostly positive) that the school counselor collected from his main classroom and “resource” teachers. His mother has said previously that she would be interested in exploring the possibility of using medications if it would help him do better in school.	Medical co-morbidity	Born prematurely, always been a picky eater; has tracked along growth at about the 10th percentile with a low but consistent ratio of weight for height.
		Difficult family	Family has always been a bit more demanding; mother feels that teachers are too quick to blame child rather than spending time helping him with work.
		Psychiatric co-morbidity	Child often says to family that he is “dumb” and would rather do things alone instead of playing with his classmates.
Anxiety	An 11 year-old boy you have followed in your practice has no chronic medical problems, though you have perhaps had more than the usual number of after-hours phone calls about concerns from his mother. This year he started middle school, and his mother is out of the home more than in the past because of a job change. He now wants a light on in his room at night, and will sometimes awaken and say that he has had a bad dream or can’t sleep because he is worrying about an upcoming school deadline. Despite all this, his school performance remains reasonable, and he still plays with friends and enjoys his other activities.	Medical co-morbidity	Has well-controlled asthma (uses mostly only a maintenance inhaler). However, in the past, he had some serious episodes and once had to be admitted to the ICU.
		Difficult family	The family has always been a bit difficult, coming late for appointments, getting behind on immunizations; mother thinks the child is just reacting to father’s more no-nonsense approach.
		Psychiatric co-morbidity	Some mornings does not want to get out of bed to go to school; trembling as said goodbye to get on the bus, wet the bed one night for the first time since toddler.
Depression	A 15 year-old girl who has been a patient in your practice since early childhood has no major medical problems and her medical transition to adolescence seems to have gone smoothly. However, partway through her first year in high school, her good grades and good mood seem to have fallen off some. This comes to light at a visit prompted by a concern for low energy and her mother wondering if she could have “mono” or Lyme disease. You talk to the patient alone and find that she is worried about her father, who has a serious illness, and that she has had trouble finding her place among new social circles in school. She says that her appetite is off, her sleep is restless, and she is spending more time to herself. However, she has no thoughts of harming herself and there is no history of self-harm in her past or in her family.	Medical co-morbidity	Has juvenile onset diabetes but with good adherence to treatment and good adjustment to having a chronic condition.
		Difficult family	Family has always seemed demanding; mother dismisses patient’s concerns about her father as “excuses” and insists on blood tests.
		Psychiatric co-morbidity	Some past history of mood fluctuation; once ran away to a friend’s house; when distressed rubs her arm with a pencil eraser until the skin is raw to “drown out” her problems; asks not to tell mother “because it will just make it worse” but has no suicidal ideation or other risk behaviors.

### Administration

The survey was administered via the Internet using a standard platform (Qualtrics, Provo, UT). A paper version of the on-line work vignette survey is available as Additional File 1. Respondents were asked to consider the vignette describing uncomplicated ADHD as a reference point and then rate the 11 others, assuming that they would be responsible for initial management and follow-up rather than making a referral. All respondents were given the vignettes in the same order: ADHD (baseline not rated, plus 3 variants which were rated, then 4 variants each of anxiety and depression which also were rated).

For each vignette, respondents were asked to rate the overall amount of work involved, assuming that the problems were newly presented by patients with whom they had a prior relationship. Respondents were asked to consider work that might need to be done before, during, and after the visit described in the vignette and in any subsequent visits. They were shown a rating scale extending from 0 to 5 with a highlight at 2 (the anchor value assigned to the reference case) and an arrow extending to the right. After each vignette, respondents were asked to write in any positive integer that they felt represented the amount of work involved in the case. The anchor of “2” and integer scale were chosen to approximate the range of relative value units (RVUs) used in US fee-for-service billing codes corresponding to commonly occurring pediatric office visits for established patients (99212, 10 min, 1.22 RVU; 99213, 15 min, 2.44 RVU; 99214, 25 min, 2.92 RVU) [29]. Practicing pediatricians are familiar with these coding options. We used “2” as the scale anchor to represent a 15 min visit, which is the average time for uncomplicated well-child visits [16].

After each overall work rating, respondents were asked to indicate whether the following were greater, less, or the same as the reference case: the total physician time involved, staff time, physical effort, mental effort, stress, and malpractice risk.

Following the vignettes, respondents answered questions about themselves, including their training and practice setting. They also completed the Physicians’ Belief Scale (PBS), a 14-item measure of attitudes toward the care of patients with psychosocial problems [30, 31]. Higher scores reflect more negative attitudes and are inversely correlated with patients’ disclosure of psychosocial information [31, 32]. The PBS has two subscales: the belief subscale includes items relating to providers’ feelings of competence to address psychosocial problems and their beliefs about patients’ desires to discuss them; the burden subscale includes items about the impact of psychosocial problems on overall workload, competing demands, and available time. A paper copy of the electronic survey is available from the corresponding author.

The survey was administered anonymously, but respondents could link to a separate Internet page where they could register for a chance to win a gift card. The study was approved by the Johns Hopkins School of Public Health IRB.

### Population

Respondents were pediatricians who self-identified as having worked in a general pediatric ambulatory setting within the last five years. They were recruited via the Maryland chapter of the American Academy of Pediatrics (AAP). First, letters were sent to members describing the purpose of the study and alerting them that they would be receiving an e-mail with a link to the survey. The chapter then used its e-mail directory to send the link; this initial e-mail was followed by three e-mail reminders.

It is not known how many chapter members were eligible for the study. The Chapter’s email list contains about 900 entries, of which about 600 are thought to be addresses of members who are actively practicing, though not all in primary care. Maryland’s Department of Labor estimates that there are about 540 pediatricians practicing primary care in the state [33]. In 2005, the AAP estimated that nationally about 80 % of pediatricians were members, [34] although estimates of specialty society membership among physicians in general range from 50-70 % depending on the specialty [35]. Thus the number of possible respondents could range from about 270 to 430.

### Analysis

Responses were downloaded directly from the survey site. We deleted partial responses (only a few initial items completed), and did not attempt to impute values for any vignettes for which work values had not been reported. For analysis, we created a data file in which each rating of a vignette was considered an individual case. Each rating was identified with a code for the respondent and dummy variables indicating diagnosis and the three cross-cutting factors.

After initial data exploration, we conducted three sets of analyses. The first addressed whether overall work ratings varied by respondent characteristics, the second set of analyses addressed the differential relationship of diagnosis and cross-cutting factors to overall work ratings, and the third addressed which components of work (total time involved, staff time, physical effort, mental effort, stress) were related to variation in the overall work ratings For each set of analyses, we began with crude bi-variate analyses and summarized results using mixed effects linear or logistic regression with random effects at the respondent level (STATA Release 12 xtmixed and xtmelogit (StataCorp, College Station, TX)) to account for clustering of responses within respondents.

## Results

1. Description of the respondents
  We received 58 responses to the vignettes, but only 48 respondents completed questions on demographic and practice characteristics, which followed the vignettes in the survey. As shown in Table 2, approximately three-quarters of respondents reporting their characteristics (*n* = 37, 77 %) were female. About half (*n* = 28, 58 %) had been in practice at their current site for 10 or more years. Similar proportions (*n* = 25, 52 %) were in urban locations and reported having a patient population whose care was predominantly insured by Medicaid. Just under half (*n* = 23, 48 %) worked with a co-located mental health provider. PBS belief scale scores had a mean of 12.6 (range 8–22, median 12); the burden scale mean was 17.4 (range 6–24, median 18).

**Table 2:** Relationship of provider characteristics to overall work ratings – bi-variate, unadjusted relationships

Provider characteristics	N (%) of total 48 or mean (SD)	Unadjusted difference in work rating (over all rated vignettes)*	95 % confidence limits for difference*
Gender (female versus male)	37 (77 %)	0	-.23 .22
At site 10 or more years versus less than 10 years	28 (58 %)	-.1	-.12 .26
Prior training in therapy or behavior (yes/no)	5 (10)%	**-.27**	**-.47 -.06**
PBS burden scale (above/below mean)	Mean 17.3 (SD 4.5)	0 (correlation r = .01, *p* = .75)	-.21 .19
PBS belief scale (above/below mean)	Mean 12.6 (SD 3.6)	**-.28** (correlation r = −.11, *p* = .01)	**-.47 -.08**
Urban practice (vs rural or suburban)	25 (52 %)	-.1	-.14 .25
50 % or more of patients in practice receive Medicaid (versus <50 %)	25 (52 %)	0	-.23 .14
Private practice vs. clinic or hospital-based	21 (44 %)	0	-.15 .22
Have co-located mental health worker versus no co-located worker	23 (48 %)	**.30**	**.11 .48**

**Fig. 1:**
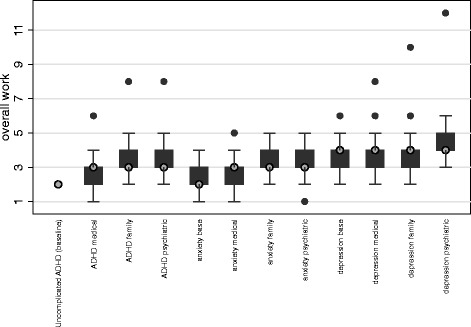
Unadjusted total work ratings by vignette. Shaded circles indicate median, boxes indicate the 25-75th percentile range, solid circles are outliers. The rating for uncomplicated ADHD was assigned as the reference and thus shows no variation
  The cross-cutting issues derived from the qualitative studies were also associated with increased work ratings compared to the reference case. Averaged across diagnoses, the unadjusted mean rating given to the complex family cases was 3.5 (95 % CL 3.4–3.7); the psychiatric co-morbidity mean was 3.7 (95 % CL 3.5–3.9), and the medical co-morbidity mean 3.0 (95 % CL 2.8–3.2). The family factor related differently to depression than to anxiety and ADHD. For both anxiety and ADHD, the difficult family factor was associated with an increase in overall work, while it was not for depression. Psychiatric co-morbidity significantly increased work for all three diagnoses, as did medical co-morbidity to a lesser extent (statistically significant for ADHD and anxiety, increased but not significant for depression) (results not shown).
  Table 3 shows changes in the number of work units accounted for by diagnosis, cross-cutting factors, and respondent factors when they are analyzed together and controlling for clustering by respondent. When diagnoses are considered alone (Model 1) anxiety is seen as only slightly but significantly more work than ADHD, but depression is greater on average by 1.2 units (or about a 60 % increase). The proportion of variance between providers was calculated from the estimated random effects parameters of a null model. The total variance was 1.266, with the proportion within providers 79.4 % and that between providers 20.6 %.

*Bold entries are significant at *p* < .05; indicates difference in estimated amount of work where 2 units was the value assigned to a reference case
2. Relationship of overall work to practice and provider characteristics:
  The 58 respondents provided 593 vignette ratings. The work ratings assigned to vignettes ranged from 1 to 12 with a modal value of 3, a mean of 3.4 and a standard deviation of 1.12 (compared to the value of 2 assigned to the reference case of uncomplicated ADHD). Nearly all (88 %; 520/593) of the assigned values were within the range of 2–4, indicating that the vignettes represented amounts of work that were similar to or greater than the reference case. Forty percent (235/593) of responses were greater than 3, but of these 71 % (168/235) were equal to 4.
  Overall crude bivariate work ratings did not differ by gender, years at practice location, urban vs. suburban/rural, percent Medicaid patients, or private practice versus other structures (Table 2). Respondents who worked with co-located mental health providers tended to give higher work ratings and those with behavior or psychotherapy training tended to give lower ratings. Respondents with PBS belief scores greater than or equal to the group mean (those who, relative to the group, had less of a psychosocial orientation) gave lower work ratings; there was no difference related to the PBS burden scale.
3. Relationship of overall work to diagnosis and cross-cutting factors:
  Unadjusted means of overall work ratings (Fig. 1) revealed that both diagnosis and cross-cutting issues influenced the amount of work respondents associated with vignettes. The uncomplicated anxiety case was rated as slightly more work (mean 2.4, 95 % CL 2.2–2.6) than the reference uncomplicated ADHD case (assigned a value of 2). The uncomplicated depression case was rated as involving substantially more work (mean 3.6, 95 % CL 3.3–3.9) than the reference case.

**Table 3:** Regression estimates (95 % CI’s) of differences in mean work ratings by condition, cross-cutting factors, and provider/practice characteristics

Parameter	Model 1^a^	Model 2^a^	Model 3^a^
Diagnosis (ADHD is reference)			
Anxiety	.18 (.03, .34)	.12 (−.01, .26)	.08 (−.069, .24)
Depression	1.21 (1.04, 1.37)	1.14 (1.01, 1.28)	1.09 (.94, 1.25)
Cross-cutting factor (simple case is reference)			
Medical co-morbidity		.49 (.33, .64)	.44 (.26, .61)
Complex family		.90 (.75, 1.06)	.87 (.69, 1.04)
Psychiatric co-morbidity		1.10 (.94, 1.25)	1.07 (.89, 1.24)
Provider/practice characteristics^b^			
Training in behavior or therapy			-.21 (−.54, .12)
Co-located MH			.24 (−.06, .55)
PBS Belief Scale > =mean			-.19 (−.50, .12)
Model statistics			
Model chi2	242.05 (*p* < .0001)	551.21 (*p <* .0001)	422.28 (*p* < .0001)
Variance and proportion of variation among respondents	.222 (23 %)	.195 (26 %)	.232 (29 %)
Number of vignettes rated	593	593	576

^a^Successive models explore work ratings as first a function only of diagnosis (Model 1), diagnosis and cross-cutting factors (Model 2) and diagnosis, cross-cutting factors, and provider/practice characteristics (Model 3)

^b^as fixed effects at same level
  When the complicating factors derived from the interview studies are added (Model 2), anxiety as a diagnosis per se no longer contributes significantly to a change in work, and the increase attributed to depression is slightly less. All of the cross-cutting factors increase work, though medical co-morbidity increases it the least (by about 25 %) and family issues and psychiatric co-morbidity the most (by about 45 and 55 %, respectively).
  When the three respondent characteristics that were significant in bivariate analyses are added (training, co-location, and PBS belief scale score) (Model 3), they have the same direction as in the crude analyses but none was significant at the .05 level and as a group they do not add significantly to the model (Wald test chi-square = 6.65, *p* = .084). Models 2 and 3 cannot be directly compared because they have different sample sizes. Calculating Model 2 using only the respondents in Model 3 allows a direct comparison: Model 2 then has a lower Bayesian Information Criterion (BIC) than Model 3 (1460.1 vs 1472.9) also suggesting that adding provider characteristics to the model does not improve prediction of work ratings.
  The proportion of within-provider variance explained by vignette characteristics was calculated by comparing the within-provider and between-person variances from Model 3 (.578 and .232, respectively) with the comparable figures from the null model. The proportion of within-provider variance explained by model 3 is 42 % and the proportion of between-person variance explained is 11 %.
4. Relationship of overall work to components of work

Table 4 shows how respondents’ estimates of physician and staff time, physical effort, mental effort, stress, and malpractice risk varied, taking all the comparison vignettes together versus the baseline vignette and adjusting for diagnosis, cross-cutting factors, and clustering among respondents. Only for physician time, mental effort, and stress were more than 50 % of the vignettes rated as requiring increased work compared to the reference case. For staff time, physical effort, and risk, more than 50 % of the vignettes were rated as requiring the same or less work than the reference case. There were statistically significant differences in these distributions for respondents working with and without co-located mental health providers. Adjusting for diagnosis, cross-cutting factors, and clustering among respondents, those working with co-located mental health providers were more likely to rate comparison vignettes as requiring increased physician time, staff time, and mental effort compared to the baseline vignette.

**Table 4:** Relationship of work components to overall work ratings, all vignettes compared to reference vignette, by respondent status (co-located with mental health provider versus not co-located)

Work components	Number (percent) of vignettes rated as more work by all respondents (*n* = 593 rated vignettes)^a^	Number (percent) of vignettes rated as more work for co-located respondents (*n* = 276 rated vignettes)^a^	Number (percent) of vignettes rated as more work for non-co-located respondents (*n* = 300 rated vignettes)^a^	Test of co-location predicting “more” versus same or less^b^
Physician time	477 (73)	217 (79)	206 (69)	.87 (*p* = .008)
Staff time	227 (35)	116 (42)	84 (28)	.99 (*p* = .039)
Physical effort	210 (34)	100 (36)	97 (32)	.13 (*p* = .85)
Mental effort	458 (71)	209 (76)	199 (66)	.69 (*p* = 0.049)
Stress	412 (63)	184 (67)	179 (60)	.53 (*p* = .21)
Risk	233 (35)	106 (38)	98 (33)	.52 (*p* = .35)

^a^Because of missing responses for co-location, the total number of vignettes rated for co-location comparisons is 576, versus 593 vignettes rated overall

^b^logistic regression coefficient adjusted for vignette diagnosis and cross-cutting factors, accounting for nesting within respondent

## Discussion

Experienced pediatric primary care providers attribute the extra work of mental health care both to diagnosis and to cross-cutting, complicating factors such as complex families and medical and psychiatric co-morbidity. Physician time, mental effort, and stress are more likely to contribute to this work compared to the efforts of other staff members or concern for malpractice risk.

The use of vignettes allowed us to differentiate these issues and may prove useful as a way to model the feasibility of integrating various forms of mental health treatment into pediatric primary care. As an example, knowing that managing depression is seen as significantly more work than routine care suggests that recent calls for the expansion of depression screening [36] may need to be paired with additional support or payment, at least initially. Pediatricians may feel that their current skill-set does not include depression-specific skills, and they may worry about self-harm, even when patients do not report those thoughts [17].

Two results suggest opportunities for promoting integration of mental health into primary care. Pediatricians attributed relatively less work to anxiety, perhaps because counseling for developmentally-related anxiety problems (such as fears of the dark and school avoidance) is already commonly taught in current pediatric training [37]. Training programs may be able to extend this existing knowledge to more pervasive or chronic anxiety problems. The relatively small amount of additional work perceived as being posed by medical co-morbidity may create an opportunity to engage pediatricians involved in or contemplating medical home efforts, but who might not otherwise have been interested in acquiring mental health skills. Medical homes focus on care of children with chronic somatic problems who may also have emotional and behavioral co-morbidities. Then, mental health expertise gained in this setting may generalize to the care of children whose problems are predominantly emotional or behavioral.

Two lines of research provide clues to how the increased work involved with cross-cutting issues could be reduced. First, training approaches for the skills involved with managing patient and family interactions have been well-received and shown to impact child outcomes [38]. Second, problem- rather than diagnosis-focused approaches to initial mental health treatment [39, 40] have helped mental health providers efficiently identify treatable concerns related to the patient and family issues presented in our vignettes, and there is preliminary evidence that they are effective in primary care [41].

The three provider characteristics that were significant in bivariate analyses (working with a co-located practitioner, having prior mental health training, and psychosocial mindedness) maintained their directionality but were not significant in the multivariate analysis of overall work. However, respondents working with co-located mental health providers were significantly more likely to say that the rated vignettes would require more physician time and mental effort, in addition to more staff time. This was unexpected, and explanations could depend on whether co-located workers were present to simply provide services independently of the pediatrician or to work collaboratively [42]. In both cases there could be more work involved in making a “warm handoff” to the co-located provider. In a more collaborative arrangement, or simply because of better communication, there could be additional pediatrician interventions triggered by the co-located provider’s suggestions. A meta-analysis of co-location programs found that without a formal attempt at collaboration, co-located mental health workers have little impact on primary care providers’ care of mental health problems (rates of referral, medication prescribing) [43].

### Limitations

This study has a number of limitations. The vignettes were based on common mental health concerns that, in pilot testing, the providers who advised us saw as common challenges. We had to depart from strictly parallel representations of baseline cases and cross-cutting factors in order to create vignettes that providers found recognizable from their experience. In primary care, concerns about anxiety and depression, for example, nearly always present in the context of environmental stressors that influence the approach to treatment. These variations could well have led to differences in perceived work that can not be attributed strictly to diagnosis. Future work could explore in more detail how various contextual factors influence treatment choices for particular diagnoses. This might strengthen our hypothesis that contextual factors drive work equally or more than the underlying condition. Future work could also involve other survey techniques such as discrete-choice/best-worst scaling, which might yield better ranking of the impact of diagnoses and factors as well as clearer links of impact to provider characteristics [44].

In addition, the factors portrayed in the 12 vignettes do not exhaust the possible combinations of diagnoses and cross-cutting factors, and the way the survey was administered did not allow combining more than one cross-cutting factor at a time, whereas in actual practice the cross-cutting factors may themselves co-occur. We also administered the vignettes in a fixed order, so that ratings could have been biased by both training effects and response fatigue. Despite having felt assured during pilot testing that the length of the survey was reasonable, the fact that some respondents did not complete the demographic and practice questions at the end of the instrument suggests that respondents did tire of the survey.

Our overall work rating scale with an anchor at 2 and integer responses was chosen because of its approximation to the numbers of RVUs assigned to common problem-oriented office visits [29]. Pediatricians must chose to bill in these discrete units. We believed that this approach would allow us to more directly understand whether variation fell within the scope of work that might be seen as practical given current financing, but it does not yield fine-grain differences in work levels. Subsequent studies could use scales anchored on larger numbers, as were the studies used in the initial development of the RVU system [27].

Another limitation is that we can say little about the representativeness of the respondents compared to practicing pediatricians. The sample is skewed to individuals with longer experience and greater familiarity with mental health either via training or work with a co-located specialist. We do not know the actual response rate for the survey, but estimate it to be between 13 and 21 %. This is small but similar to the response rate (26 %) reported for email-only administration of a survey to Georgia pediatricians [45].

Many of these limitations may be addressed by combinations of different means of administration. A shorter survey, randomly allocating vignettes to a larger and more representative group could increase response rate. Mail administration would likely have a higher response rate, [45] or offering an incentive to each respondent rather than a lottery. Endorsement by a payer or professional organization noting that the results could be used to develop new compensation schemes might further incentivize response.

## Conclusions

This study suggests a practical method for planning task shifting mental health interventions into pediatric primary care. Ratings of vignettes by experienced providers can help differentiate the work providers attribute to a particular diagnosis from work attributable to other clinical factors. Asking in this fashion may yield more information than surveys simply asking about clinicians’ willingness to take on additional work related to mental health. Our study also provides preliminary evidence that some of the factors that contribute to the work of providing mental health services in primary care cut across diagnoses and may thus be addressed efficiently through both provider and system-level interventions. Initial approaches may best target diagnoses or patient groups that at present are seen as involving feasible levels of work. Building on this base, more daunting diagnoses may eventually be seen as approachable in primary care.

## Additional file

Additional file 1:
**Paper version of on-line work vignette survey**. (PDF 84 kb).
