# Supplementary material for: Using vignettes to assess contributions to the work of addressing child mental health problems in primary care
Source: BMC Health Serv Res. 2016 Jan 22;15:584. doi: 10.1186/s12913-015-1237-x (PMC4722679; doi:10.1186/s12913-015-1237-x)
Supplement: Additional file 1: — Paper version of on-line work vignette survey. (PDF 84 kb). [file 12913_2015_1237_MOESM1_ESM.pdf]

[Print version of web-based survey]

PRIMARY MENTAL HEALTH CARE WORKLOAD QUESTIONNAIRE

CENTER FOR MENTAL HEALTH SERVICES IN PEDIATRIC PRIMARY CARE

Johns Hopkins School of Public Health & University of Maryland

Dear Colleague: We are trying to learn more about the amount of work involved in evaluating, treating, and referring children seen in primary care who may have mental health problems. Our goal is to develop tools to measure this kind of work so that it can be properly compensated and, if possible, made easier. After talking with a number of clinicians, we developed a set of clinical cases that seem typical of those often presenting in primary care practices. You will be asked to review the cases and answer a few questions about yourself and your practice. The survey should take approximately 10 minutes to complete. Some of the questions about practice composition and compensation could be considered sensitive business information, but we do not ask for any information that would identify your specific practice.

SCREENING QUESTIONS

Please select the option that best describes your role over the past 5 years:

- ❍ During the past 5 years, I have worked as a clinician in a general ambulatory pediatric practice setting (e.g., a pediatric primary care practice or clinic) (1)
- ❍ I have not been in active clinical practice within the past 5 years (2)
- ❍ None of my clinical work during the past 5 years has been in a general ambulatory pediatric setting (3)

INSTRUCTIONS

The relative value unit (RVU) scale (shown below) indicates the total value of primary care provider work required to manage a newly emerging condition until the condition resolves or until 12 months have elapsed, whichever occurs first. Your rating should thus include work that might need to be done before, during, and after this initial visit and any subsequent visits. “Work” includes:

- The actual time it would take you to care for this patient
- The degree of technical skill and physical effort required
- The mental effort involved (e.g., patience, concentration, motivation, persuasiveness, flexibility, empathy and judgment)
- The psychological stress associated with clinical responsibility and concern for the patient.

Some vignettes may indicate the need for a greater number of appointments (i.e., more time) whereas others may indicate greater mental effort and/or stress around eventual collaboration with specialists and/or parents. Please also consider any additional tasks you may need to complete, such as ruling out physical explanations for a child’s symptoms or additional

paperwork that may be needed. The cases are similar but involve variations that may alter the amount of work involved. We are interested in a very general estimate based on your overall impression from the limited information provided.

The RVU scale is calibrated such that all points on the scale represent a workload value compared to the reference case, which is pre-assigned an RVU workload rating of 2. Your ratings may be any positive integer value. For example, a case that requires twice as much work as the reference case would get a rating of 4; a case that requires half as much work would get a rating of 1.

Here is the case we are using as the reference (RVU=2)

An 8 year-old boy, who has been a patient of yours for several years, has no known developmental issues, no chronic health problems, and lives with a stable, well-functioning family. He has long had moderate academic difficulty in school despite good effort; today he comes with his mother who has brought a packet of Vanderbilt forms (mostly positive) that the school counselor collected from his main classroom and “resource” teachers. His mother has said previously that she would be interested in exploring the possibility of using medications if it would help him do better in school. You take a history from parent and child, prescribe stimulant medication, and provide brief counseling on medication use and side effects. The patient’s care for this condition is subsequently managed by you (not referred out to a psychiatrist).

CASES FOR RESPONDENT RATING

Case A1. Using the reference case, consider an 8 year old boy who was born prematurely. He has always been a picky eater; he has tracked along growth at about the 10th percentile with a low but consistent ratio of weight for height. You take a history from parent and child, prescribe stimulant medication, and provide brief counseling on medication use and side effects. The patient's care for this condition is subsequently managed by you (not referred out to a psychiatrist).

1. Compared to the reference case (RVU=2), what is your overall RVU rating for this case:
2. How does this case compare to the reference case in relation to…?

	Less (1) (1)	Same(2) (2)	More(3) (3)
a. total amount of primary care provider time required (1)	❍	❍	❍
b. other staff time, practice overhead, and/or materials (2)	❍	❍	❍
c. physical effort required (3)	❍	❍	❍
d. mental effort (e.g. determine a diagnosis, management of treatment, ascertaining if the patient is improving etc.) (4)	❍	❍	❍
e. psychological stress (e.g. worry about risk to patient) involved (5)	❍	❍	❍
f. malpractice risk (6)	❍	❍	❍

Case A2. Using the reference case, consider an 8 year old boy who has a family that has always been a bit more demanding than other families in your practice, coming late for appointments, getting behind on immunizations and well-child care, and sometimes coming for an acute visit but then not following through with the recommended treatment. You take a history from parent and child, and, after talking about the diagnostic possibilities, prescribe stimulant medication and provide brief counseling on medication use and side effects. The patient's care for this condition is subsequently managed by you (not referred out to a psychiatrist).

[all cases have same response questions as for Case A1 so responses are not repeated]

Case A3. Using the reference case, consider an 8 year-old boy who has been tearful every time something does not go well in school. Despite the patient's parents' best efforts to encourage him, he increasingly tells them that he is "dumb" and would rather do things alone instead of playing with his classmates. You take a history from parent and child, and then, after talking about the diagnostic possibilities, prescribe stimulant medication and provide brief counseling on medication use and side effects. The patient's care for this condition is subsequently managed by you (not referred out to a psychiatrist).

Case B1. An 11 year-old boy you have followed in your practice has no chronic medical problems, though you have perhaps had more than the usual number of after-hours phone calls about concerns from his mother. This year he started middle school, and his mother is out of the home more than in the past because of a job change. He now wants a light on in his room at night, and will sometimes awaken and say that he has had a bad dream or can't sleep because he is worrying about an upcoming school deadline. Despite all this, his school performance remains reasonable, and he still plays with friends and enjoys his other activities. You briefly review the family situation and recent health history. You then provide brief counseling and psychoeducation about transient anxiety problems and discuss a plan for the parent to re-contact you if the problem persists.

Case B2. Now consider another 11 year old boy with sleeping problems similar to case B1, but who also has well-controlled asthma (uses mostly only a maintenance inhaler). However, in the past, he had some serious episodes and once had to be admitted to the ICU. You briefly review the family situation and recent health history. You provide brief counseling and psychoeducation about transient anxiety problems and discuss a plan for the parent to re-contact you if the problem persists.

Case B3. Consider another 11 year-old boy you have followed in your practice with sleeping problems similar to case B1 but with no chronic medical problems, though you have perhaps had more than the usual number of after-hours phone calls about concerns from his mother. In addition, the family has always been a bit difficult for your practice, coming late for appointments, getting behind on immunizations and well-child care, and sometimes coming for an acute visit but then not following through with the recommended treatment. His mother tells you that she thinks the problem is that he's just always been a "mommy's boy" and is reacting to the fact that his father takes a more no-nonsense approach to homework and household chores. You briefly review the family situation and recent health history. You provide brief

counseling and psychoeducation about transient anxiety problems, talk about parental agreement on child discipline, and discuss a plan for the parent to re-contact you if the problem persists.

Case B4. Consider another 11 year-old boy you have followed in your practice with sleeping problems similar to case B1 and no chronic medical problems. Some mornings he has said he doesn't want to get out of bed to go to school, and once he seemed even to be trembling as he said goodbye to get on the bus. This visit was prompted because he had wet the bed one night last week, something that had not happened since he was a kindergartner. You briefly review the family situation, recent health history, and school environment. You provide brief counseling and psychoeducation about transient anxiety problems, and discuss a plan for the parent to re-contact you if the problem persists.

Case C1. A 15 year-old girl who has been a patient in your practice since early childhood has no major medical problems and her medical transition to adolescence seems to have gone smoothly. However, partway through her first year in high school, her otherwise good grades and good mood seem to have fallen off some. This comes to light at a visit prompted by a concern for low energy and her mother wondering if she could have "mono" or Lyme disease. You talk to the patient alone and find that she is worried about her father, who has a serious illness, and that she has had trouble finding her place among the new social circles in school. She says that she's found her appetite to be off, feels that her sleep is restless, and she is spending more time to herself. However, she says that she has no thoughts of harming herself and there is no history of self-harm in her past or in her family. You take a history separately from mother and child, and conduct a brief physical examination of child. You also briefly counsel mother and child about coping with school stresses and the family's challenges surrounding her father's illness, discuss possible laboratory tests for medical conditions that can cause fatigue and mood changes, and schedule a follow-up visit.

Case C2. Now consider another 15 year old girl in your practice describing fatigue and mood changes similar to case C1 who also has juvenile onset diabetes with good adherence to treatment and good adjustment to having a chronic condition. She tells you that she continues to be "fine" with her diabetes management. You take a history separately from mother and child, and conduct a brief physical examination of child. You also briefly counsel mother and child about coping with school stresses and the family's challenges surrounding her father's illness, discuss possible laboratory tests for medical conditions that can cause fatigue and mood changes, and schedule a follow-up visit.

Case C3 Now consider another 15 year old girl in your practice describing fatigue and mood changes similar to case C1 whose parents you have always experienced the parents as very demanding. With the girl's permission, you share the girl's concerns about her father's illness and school stressors with her mother, who immediately dismisses them as "excuses" and asks again about blood tests. You take a history separately from mother and child, and conduct a brief physical examination of child. You also briefly counsel mother and child about coping with school stresses and the family's challenges surrounding her father's illness, discuss possible

laboratory tests for medical conditions that can cause fatigue and mood changes, and schedule a follow-up visit.

Case C4. Please consider a 15 year-old girl similar to case C1. Over the years, you have worked with her on mood and behavior issues that have been somewhat outside the range of normal but have not greatly interfered with her function. You talk to the patient alone and find that she has had marked loss of appetite and interest in school for over a month and poor sleep. She has had some vague thoughts of wishing she could just escape and once “ran away” to a friend’s house overnight, but convincingly says that she has no suicidal ideation or plan. When she feels distressed, she will retreat to her room and rub her arms with a pencil eraser until the skin is raw – she says that this makes her feel better and “drowns out” her problems. When you ask if you can share these concerns with her mother, she asks you not to because it will just make matters worse. You take a history separately from mother and child, and conduct a brief physical examination of child. You also briefly counsel mother and child about coping with school stresses and the family’s challenges surrounding her father’s illness, discuss possible laboratory tests for medical conditions that can cause fatigue and mood changes, and schedule a follow-up visit.

CLINICIAN BACKGROUND AND PRACTICE CHARACTERISTICS

A. Clinician Information
Your degree:

- ❍ DO (1)
- ❍ MD (2)
- ❍ NP (3)
- ❍ PA (4)
- ❍ Other (5) ____________________

In what year did you receive your degree?

Your primary specialty (check all that apply):

- ❑ Family practice (1)
- ❑ Pediatrics (2)
- ❑ Internal medicine (3)
- ❑ Other Specialty (please write in): (4) ____________________

Except for the basic curriculum for your degree and residency (or other required post-degree training), have you had any specialized training (workshop, short course, fellowship, etc.) in any of the following? (check all that apply):

- ❑ Child behavior (1)
- ❑ Child development (2)
- ❑ Counseling or psychotherapy (3)
- ❑ No specialized training in child behavior, development, or psychotherapy (4)

How long have you been (or were you) a clinician at your present (or most recent) practice site? (If you practice(d) at more than one site, answer for the site you consider(ed) your primary site)

- ❍ Less than a year (1)
- ❍ 1-4 years (2)
- ❍ 5-9 years (3)
- ❍ 10-14 years (4)
- ❍ 15 or more years (5)

Your gender:

- ❍ Male (1)
- ❍ Female (2)

B. Practice Information
In what type of organization do you currently or did you previously practice? (choose one answer - if you practice(d) at more than one site, answer for the site you consider(ed) your primary site)

- ❍ HMO (1)
- ❍ FQHC/Public clinic (2)
- ❍ Free-standing private practice (3)
- ❍ Hospital or hospital-owned ambulatory primary care facility (4)
- ❍ Other (please write in): (5) ____________________

What is/was the structure of your practice?

- ❍ Single speciality (1)
- ❍ Multi-specialty group (2)

Is/was there a psychologist, social worker, or other mental health specialist on site?

- ❍ No (1)
- ❍ Yes (2)

Which best describes/described your primary practice setting? (choose one answer)

- ❍ Rural (1)
- ❍ Suburban (2)
- ❍ Urban (3)

Approximately what proportion of all your patients is/was enrolled in Medicaid or the Children's Health Insurance Program (CHIP)?

- ❍ 0% to 24% (1)
- ❍ 25% to 49% (2)
- ❍ 50% to 74% (3)
- ❍ 75% to 100% (4)
